# Possible identification of CENP-C in fish and the presence of the CENP-C motif in M18BP1 of vertebrates.

**DOI:** 10.12688/f1000research.6823.2

**Published:** 2016-01-20

**Authors:** Leos Kral

**Affiliations:** 1 Department of Biology, University of West Georgia, Carrollton, GA, 30118, USA

**Keywords:** CENP-C, M18BP1, centromeric proteins, teleostei, kinetochore, CENP-C motif, cupin domain protein

## Abstract

The centromeric protein CENP-C is a base component of the kinetochore. This protein, along with CENP-A has been shown to adaptively evolve in a number of animal and plant species. In order to determine if CENP-C also evolves in fish species, I attempted to retrieve fish CENP-C sequences from GenBank. No Teleostei CENP-C sequences were found either by name or by BLASTP searches with the vertebrate CENP-C motif sequence. A number of putative Teleostei protein sequences were identified in GenBank that have homology to the C-terminal cupin domain of vertebrate CENP-C. These proteins only have partial homology to the CENP-C motif, but evidence is presented that makes it likely that these fish proteins are orthologs of CENP-C. Interestingly, it was also discovered that the CENP-C motif sequence is also mostly present in M18BP1 proteins of fish and some other vertebrates but not in mammals. This finding may have implications for CENP-C and M18BP1 assembly in centromeric regions of different vertebrate taxa.

## Introduction

The kinetochore is a structure that connects chromosomal centromeric DNA to microtubules during mitosis and meiosis ^1^. The centromere is epigenetically defined by the deposition of nucleosomes that contain the histone H3 variant CENP-A ^2^. Centromeric protein CENP-C is required for both the recruitment of new CENP-A to the centromeric region as well as the initial assembly of the kinetochore. The CENP-C protein is generally considered to be ubiquitous in all eukaryotic taxa since homologs of CENP-C have been identified in yeast ^3^ and *Drosophila* ^4^ as well as many plants and vertebrates ^5^. While CENP-C evolves so rapidly that very little homology is observed between distantly related taxa, a conserved CENP-C motif has been identified across all lineages studied ^5^. This conserved motif should, therefore, be of utility to identify CENP-C orthologs in other species.

CENP-A has been initially shown to evolve adaptively in *Drosophila* ^6^, in members of the Bressicaceae family ^7^ and more recently in primates ^8^ and in percid fishes ^9, 10^. CENP-C has also been shown to evolve adaptively in a number of animal and plant species ^5^ as well as in primates ^8^. In an effort to determine if CENP-C also evolves adaptively in fish species, searches were conducted in GenBank for Teleostei proteins that had been already identified as CENP-C or for genes that had been annotated as coding for CENP-C. No such teleost fish proteins or genes were found. BLASTP searches of just the Teleostei subset of GenBank were performed with the conserved vertebrate CENP-C motif and these too failed to find identified fish CENP-C proteins or genes. However, a set of orthologous C-terminal cupin domain containing genes have been identified in the elephant shark *Callorhinchus milii* and several teleost fish species that, while lacking most of the conserved CENP-C motif, have features that make these likely to be fish CENP-C orthologs.

## Methods

Standard BLASTP searches were performed on the NCBI blast server. The vertebrate CENP-C motif NVRRTKRXRLKPLEYWRGERVBY used in BLASTP searches in this study was obtained from an alignment of 25 species including the lobe-finned fish *Latimeria chalumnae*, amphibians, reptiles, birds and mammals ( Supplementary File S1). Retrieved sequences were aligned with the MUSCLE alignment feature in Geneious (version 6.1) sequence analysis software.

## Results and discussion

**Figure 1.**
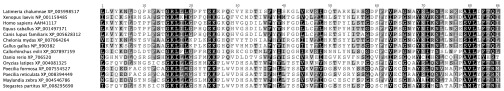
Homology of shark and teleost fish putative CENP-C cupin domains to the cupin domains of other vertebrate CENP-C proteins. Lobe-finned fish: *Latimeria chalumnae*. Amphibian: *Xenopus laevis*. Mammals: *Homo sapiens*, *Equus caballus*, *Canis lupus familiaris*. Reptile: *Chelonia mydas*. Bird: *Gallus gallus*. Shark: *Callorhinchus milii*. Teleost fish: *Danio rerio*, *Oryzias latipes*, *Poecilia formosa*, *Poecilia reticulata*, *Maylandia zebra*, *Stegastes partitus*.

**Figure 2.**
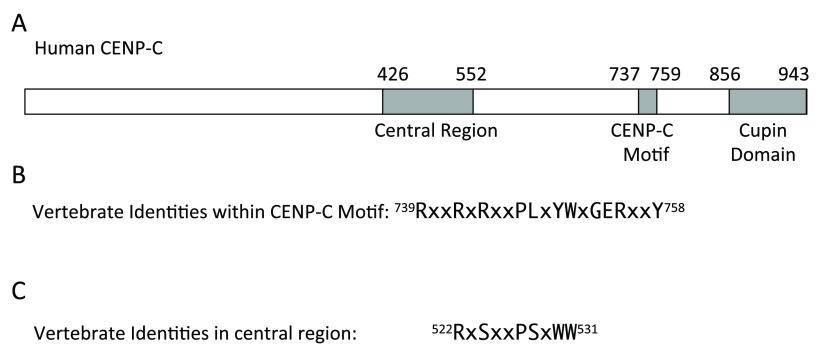
Conserved domains and sequences in vertebrate CENP-C. ( **A**) Diagram of human CENP-C. ( **B**) Amino acids that are identical in the CENP-C motif in vertebrates in which CENP-C has been identified. ( **C**) Conserved sequence in the CENP-C central region that is homologous to part of the CENP-C motif. Amino acid locations within human CENP-C protein of conserved sequences are indicated at the beginning and end of each sequence.

**Figure 3.**
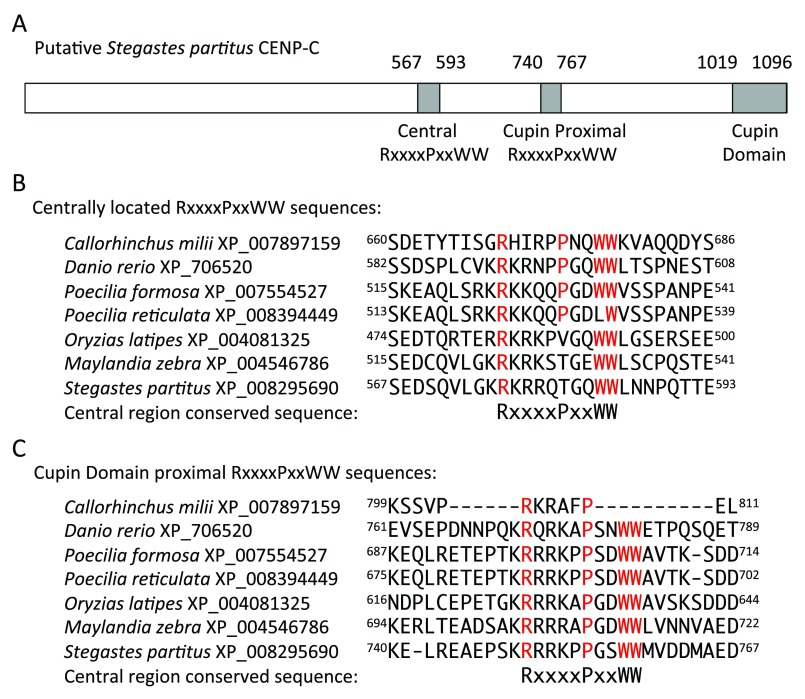
Conserved regions homologous to a portion of the CENP-C motif present in putative non-lobe-finned fish CENP-C. ( **A**) Diagram of putative *Stegastes partitus* CENP-C. ( **B**) Alignment of fish central region sequences that contain the conserved RxxxxPxxWW sequence. ( **C**) Alignment of the fish cupin domain proximal sequences that contain the conserved RxxxxPxxWW sequence. Amino acids matching the conserved sequence identities are highlighted in red. Amino acid locations within each species’ protein are indicated at the beginning and end of each sequence.

BLASTP searches with the vertebrate CENP-C motif identified CENP-C proteins from a variety of taxa, including plants, but did not identify any CENP-C in non-lobe-finned fish lineages. It is possible that CENP-C may be absent in ray-finned fish, but the ubiquity of this protein in other lineages and the central role of this protein in centromeric function make this unlikely. A C-terminal cupin domain protein encoded by a shark gene annotated in GenBank as CENP-C was used to identify homologs in Teleostei genomes by BLASTP. The retrieved teleost fish homologs were annotated as either calponin homology domain containing protein, neurofilament heavy polypeptide-like protein, or myb-like protein. The cupin domains of these proteins share significant homology to the cupin domain of other vertebrate CENP-C proteins ( Figure 1). Within vertebrate CENP-C proteins the RxxRxxxxPLxYWxGERxxY sequence defines identities within the CENP-C motif located within about 100 amino acids upstream of the cupin domain ( Figure 2). However, within the shark and teleost fish C-terminal cupin domain-containing protein sequences, only some of these CENP-C motif sequence identities were present ( Figure 3) and, therefore, unambiguous identity of these proteins as CENP-C was not obvious.

In a recent study that examined the interaction between CENP-C conserved domains and CENP-A containing nucleosomes (or nucleosomes containing histone H3 modified with a CENP-A C-terminal tail), Kato *et al.* ^11^ identified within the conserved central region of CENP-C a RxSxxPSxWW consensus sequence ( Figure 2) that is similar to the core portion of the CENP-C motif. Mutations of the arginine to alanine or the tryptophans to alanine in this sequence prevented the binding of this central region to the nucleosomes. So, functionally, the RxxxxPxxWW portion of the central region sequence is important to centromeric binding of CENP-C. Furthermore, mutations of the arginine, tyrosine and tryptophan in the core CENP-C motif RxxxxPxxYW also reduce the binding affinity the CENP-C to the nucleosomes ^11^. A mutation of arginine to alanine in this core portion of the CENP-C motif was previously shown to prevent the binding of *Xenopus* CENP-C to centromeres ^12^.

An alignment of the putative shark and teleost fish CENP-C proteins identified two conserved regions that contained the RxxxxPxxWW sequences ( Figure 3). The placement of these sequences corresponds roughly to the locations of the central portion and the CENP-C motif of the vertebrate CENP-C ( Figure 2). Therefore, it is likely that the combination of the C-terminal cupin domain and the presence of these centromeric nucleosome binding regions in positions generally corresponding to the locations of the central region and the CENP-C motif identifies these teleost genes as possible CENP-C orthologs. It will be necessary, of course, to verify if this protein is actually found at non-lobe-finned fish centromeres. It should be noted, however, that the distance between the cupin domain and the “CENP-C motif” position is about twice as long in the putative teleost fish CENP-C in comparison to this distance in CENP-C of other vertebrates. It is interesting that the putative shark “CENP-C motif” location lacks the tryptophans of the RxxxxPxxWW sequence and that *Poecilia reticulata* has a replacement of the first tryptophan in the conserved central region sequence ( Figure 3). However, depending on other factors acting in the assembly of the centromere in various taxa, it may be possible that just one of those conserved RxxxxPxxWW sequences may be necessary for centromeric binding of the putative non-lobe-finned fish CENP-C. Indeed, the conserved central region is not present in chicken CENP-C ^13^ and no homology to the RxxxxPxxWW portion of the conserved central region is detectable in CENP-C of other birds and reptiles. Yet a deletion mutant of chicken CENP-C in which the central portion had been removed was able to rescue CENP-C deficient chicken cells and also co-localized with CENP-T at centromeres ^13^. This demonstrates that, at least in chickens, the central region is not necessary for CENP-A binding and, presumably, the C-terminal region which contains the CENP-C motif is sufficient for that purpose.

**Figure 4.**
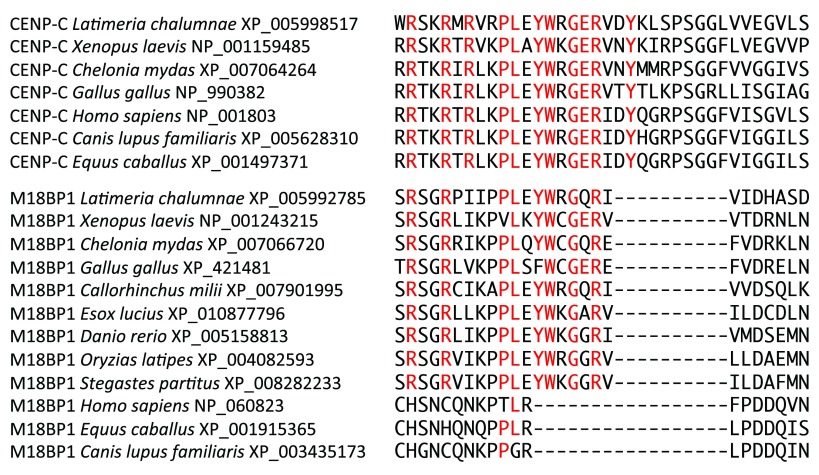
Alignment of regions of vertebrate CENP-C and M18BP1 that contain the CENP-C motif sequences. The vertebrate CENP-C motif sequence identities ( Figure 2A) are highlighted in red.

Interestingly, BLASTP searches of the Teleostei subset of GenBank retrieved centromeric protein M18BP1 sequences. This protein is recruited to centromeres by CENP-C ^14, 15^ and along with centromeric proteins Mis18α and Mis18β functions in the recruitment of CENP-A to centromeres ^16^. The M18BP1 protein contains almost the entire vertebrate CENP-C motif in all vertebrates examined except in mammals ( Figure 4). It appears that the CENP-C motif sequence is not exclusive to just CENP-C. Since both CENP-C and M18BP1 associate with centromeres and with each other, it is tempting to speculate that what has generally been regarded as a CENP-C motif sequence facilitates the interaction of both of these proteins with centromeric nucleosomes. Furthermore, since mammalian M18BP1 lacks this CENP-C motif, it is possible that mammalian M18BP1 may be more dependent on association with CENP-C to localize to the centromere than the M18BP1 of other vertebrate taxa.

## Supplementary material

**Supplementary File S1. Alignment of the C-terminal portion of vertebrate CENP-C proteins.**

The vertebrate CENP-C motif containing consensus sequence utilized in BLASTP searches spans amino acids 11 to 33 and is highlighted in red.
