# Supplementary material for: Possible identification of CENP-C in fish and the presence of the CENP-C motif in M18BP1 of vertebrates
Source: F1000Res. 2016 Jan 20;4:474. Originally published 2015 Aug 5. [Version 2] doi: 10.12688/f1000research.6823.2 (PMC4830207; doi:10.12688/f1000research.6823.2)
Supplement: Supplementary file 1 [file f1000research-4-8419-s0000.tgz › 030581f5-5f89-4723-b7de-09e6496a639a.pdf]

```
                                   1                                                50
Consensus                          HKLVLPSNTPNVRRTKRXRLKPLEYWRGERVDYXXRPSGGFVIGGILSP-
Latimeria chalumnae XP_005998517   K.II...T....W.S..M.VR...........KLS....L.VE.V...V
Xenopus laevis NP_001159485        ..IIP..K......S..T.V...A..K....N.KI......LVE.VVP.A
Mus musculus NP_031709             ........S.....SN.I...............QESS..QL.LE-.I..-
Elephantulus edwardii XP_006902789 S.....D.........T.....Q.....I..KA..............V
Chinchilla lanigera XP_005392894   Y........G......I.S.............QET.............-
Fukomys damarensis XP_010609490    ................I.S.............QET....L........-
Canis lupus familiaris XP_005628312 ................T...........I..HG..............-
Felis catus XP_006931147           ..........D.....T...........I..HG..............-
Ursus maritimus XP_008706924       ................T..............HG..............-
Equus caballus XP_001497371        Y.I.............T...........I..QG..............-
Pteropus vampyrus XP_011358951     Q...............T..............QG..............-
Orcinus orca XP_012388019          ..............M.T.S.........I..QG..............-
Camelus dromedarius XP_010992993   ..............V.T.S.........L..QG..............-
Tarsius syrichta XP_008069341      ................I...........I..QET....Y........-
Homo sapiens AAH41117              ................T...........I..QG........S.V...-
Alligator sinensis XP_006027584    ....K...........T.............N.MS........A.VVR.-
Python bivittatus XP_007423311     ....W.T.........I.I.............KT.A.........V..-
Anolis carolinensis XP_008111248   R.....T.........M.I............K.RT.......FH..I..E
Chelonia mydas XP_007064264        ....M..Q........I.............N.MM.......V...V..-
Chrysemys picta bellii XP_008171491 ....M..Q........I.............N.MM.......V...V..-
Gallus gallus NP_990382            Q.I.............I.............T.TLK...RLL.S..AGA-
Meleagris gallopavo XP_010708197   Q.I.............I.............T.TLK...RLL.S..AGA-
Charadrius vociferus XP_009879443  R.I.M...........I.............N.TMG....LM.S..VC.-
Cariama cristata XP_009693060      ..I.M.T.........I..R..........N.TM.....LM.S..VC.-
Egretta garzetta XP_009647475      ..I.M...........I..R..........N.TM.....L..S..VC.-

                                  51                                               100
Consensus                          XTXXXKXKXKXXXXXKVXK---XKXXXLXXXEXKXXXXXNL-BIPLGDPXQ
Latimeria chalumnae XP_005998517   GQQIQRRIKLRRNAERRR----------EG.GGSSAVQ..SGCSEVESTS
Xenopus laevis NP_001159485        EVEPPRKAVAKRRIYNKQ--------------.GHIPTYV-EPLTE.YVE
Mus musculus NP_031709             SSVPT.I.AQRNLG..N.KVTK.PTH.NSH.-.AKMELP.-DMR....F.
Elephantulus edwardii XP_006902789 V.LKEEKAANKETR..N.ITNR.RIC.DER.GINRILA.V-N......S.
Chinchilla lanigera XP_005392894   E.VTS.R.A.GNVE..N.IHNR.RIC.DNV.R.SRLVV.C-D......F.
Fukomys damarensis XP_010609490    E.VIS.R.A.GNVG.DN.IDNR.RIC.DNV.K.SKLVV..-G......F.
Canis lupus familiaris XP_005628312 D.VSS.T.S.GNVG..K.MANR.RIC.DDE.R.KKFMV..-N......L.
Felis catus XP_006931147           D.VSS.R.A.GNIG..N.LVNR.RIC.DND.R.NKFML..-NV.....L.
Ursus maritimus XP_008706924       D.VSS.K.S.GNIGT.S.MVNR.RIC.DNE.R.NKFLV..-S......L.
Equus caballus XP_001497371        D.ISS.R.A.GSME..NEIVNR.RIC.DNN.R.NKLVV..-N......L.
Pteropus vampyrus XP_011358951     D.VSS.T.A.GETK..N.IVNR.RIC.DND.R.EKLVV..-N......L.
Orcinus orca XP_012388019          DKVSS.R.A.GNLGR.NTIVNR.RIC.DND.R.NKFVE..-N......L.
Camelus dromedarius XP_010992993   D.VSS.R.A.ENLG..NAVDNR.RIC.DND.R.NKLVV..-N......L.
Tarsius syrichta XP_008069341      D.VLP.R.A.ENIE..K.---G.KIC.DND.R.TRLIV..-D......L.
Homo sapiens AAH41117              D.ISS.R.A.ENIG..N.KSNK.RIC.DND.R.TNLMV..-G......L.
Alligator sinensis XP_006027584    --VSPEKVK.KRNHH.Q.-------------K.HDRVED.-DVS.A.ASK
Python bivittatus XP_007423311     EQREPRKPKPK----.TR----------KSVPEMENMHDN-SVS.R..S.
Anolis carolinensis XP_008111248   QKEPI.P.S.KTTNP.ME------------------LD.MPESS.Q..P.
Chelonia mydas XP_007064264        EKDSCRKARVKRKHTPKT--------------RSHVAEH.-NVS.A.TSK
Chrysemys picta bellii XP_008171491 EKDPC.KASVKRKHTPKT--------------RHHVVEY.-NVS.A.TSK
Gallus gallus NP_990382            EAEPHMKNKLKSRH.KKR-----------AKTRSEVVKC.-D...A.ASK
Meleagris gallopavo XP_010708197   EAEPHVKNKQKSHH.QKR-----------ARTRSEVAKC.-D...A.ASK
Charadrius vociferus XP_009879443  E.GPQRQIKQRSDGDKQQ----------RC.TRSDIPA..-NHI.A.ASK
Cariama cristata XP_009693060      E.EPYRRIKRRKGGHKQ.----------RD.TRSEIPV..-DHT.A.TSK
Egretta garzetta XP_009647475      EAEPRRMIKRRKGGHKQ.----------KA.TRSEIPA..-DHT.A.TSK

                                  101                                              150
Consensus                          PTXVXDPETXEXXLMDLVRPXDTXXFF-----XEXXXLKVYKXLDTPXFS
Latimeria chalumnae XP_005998517   .VE.W.ATRSQRI.LNCM.SGNDCL.HRGTVEENNGY.L...N..Q.E.A
Xenopus laevis NP_001159485        .AK.L.AD.GTITTI.C..TSENCLYH-----DPDQPISIC.SIRNSA..
Mus musculus NP_031709             A.LAK....A.LVP...I..R..YR..-----V.QHG...F.T...IY..
Elephantulus edwardii XP_006902789 ..L.K....R.SIF.E.I..R..CQ..-----F.CKE...H.I....F..
Chinchilla lanigera XP_005392894   ..Q.K....R.II....I..R..YQ..-----V.HGE.....T....F..
Fukomys damarensis XP_010609490    ..Q.K....R.IIF...I..R..YQ..-----V.HGK.....T....L..
Canis lupus familiaris XP_005628312 ..R.K....R.IVT...L..R..YE..-----I.HDE.....T....L..
Felis catus XP_006931147           ..R.K....R.VI....L..R..YQ..-----V.HGE.....T....I..
Ursus maritimus XP_008706924       ..R.K....R.IVP...L..R..FQ..-----V.HGD.....T....I..
Equus caballus XP_001497371        ..RIR....R.II.......R..YQ.I-----V.HGE.....T....F..
Pteropus vampyrus XP_011358951     ..R.K....RKMI....L..R..YQ.S-----V.HGE.....T....L..
Orcinus orca XP_012388019          ..R.K....R.II.......R..YQ.C-----V.HGE.....T....F..
Camelus dromedarius XP_010992993   ..R.K....R.II.......R..YQ.C-----V.HGE.....T....F..
Tarsius syrichta XP_008069341      .SR.K....K.II....I..R..YQ..-----VKHGE.....S....L..
Homo sapiens AAH41117             ..R.K....R.II.......Q..YQ..-----VKHGE.....T....F..
Alligator sinensis XP_006027584    ..A.W..AVNGEVFLECISTGHNHS..-----FKDESVEI..H.N.SV.A
Python bivittatus XP_007423311     .AV.F.KASNQEI.LEC.NNGSSHV..-----IGNEAVSI..Y.S..S..
Anolis carolinensis XP_008111248   .AV.F.V.SNQGV.LQC.NTGTSHLH.-----INNEAVSI..Y.T..S..
Chelonia mydas XP_007064264        ..A.W..TIN.EV.LEC.NTGSNHSC.-----FNDESVEI..N.N.SV.A
Chrysemys picta bellii XP_008171491 ..A.W..TIN.EV.LEC.NTGRNHSC.-----FNDESVEI..N.N.SV.A
Gallus gallus NP_990382            ..SIV..V.NQEV.LECINSGSSHSC.-----F.DESI....S.N.SD.A
Meleagris gallopavo XP_010708197   ..SIV..V.NREV.LEC.NSGSSHSC.-----F.DESI....S.N.SV.A
Charadrius vociferus XP_009879443  ..P.L..V.NKEV.LEC.NTESSHTCV-----FRDETVE...N.N.SA.A
Cariama cristata XP_009693060      ..I.L..V.NKEV.LEC.NTGSSHSC.-----FKDESVEI..N.N..A.A
Egretta garzetta XP_009647475      ..I.L..V.N.EV.LEC.NTESRHTC.-----FKDESVEI..N.N.SA.A

                                  151                                              200
Consensus                          TGKLILGPXXEKGKQHVGXDTLVFYVXXGXLLCTLHETXYXLTTGDXFYV
Latimeria chalumnae XP_005998517   ..R.L...FT..KP.C.YE..IS.FISK.LVEVSIDQ.ARK.RS..F.F.
Xenopus laevis NP_001159485        ....KI..FQ...L.F.CM..I...IMT.SVQL...LST.N.K...F..I
Mus musculus NP_031709             ....V...YE........Q.I.....NF.D........P.K.....S...
Elephantulus edwardii XP_006902789 ........YQ........L...I.F.NF.K........P.LIS...S...
Chinchilla lanigera XP_005392894   ........YE........E.I.....NV.D........P.TI....S...
Fukomys damarensis XP_010609490    ........YE........E.I.....NV.D........P.AI....S...
Canis lupus familiaris XP_005628312 ........HQ........S.I.....NF.D........S.II.A..S...
Felis catus XP_006931147           M.......HQ........S.I.....NF.D........P.II....S...
Ursus maritimus XP_008706924       ........HQ........S.I.....NF.D........P.II....S...
Equus caballus XP_001497371        ........HQ........S.I.....NF.D........P.MI....S.F.
Pteropus vampyrus XP_011358951     ....V...HQ........L.......DF.D........P.IV....S...
Orcinus orca XP_012388019          ........HQ........L...I...NF.D........P.VI....S...
Camelus dromedarius XP_010992993   ........HQ........L.......NF.D........P.II....S...
Tarsius syrichta XP_008069341      ........HQ........R.I...F.NF.D........P.II....F...
Homo sapiens AAH41117              ........QE........Q.I.....NF.D........P.I.S...S...
Alligator sinensis XP_006027584    ......K.LK...Y.FIHM.KIA.H.VH.RVIF....MS.H.S...F...
Python bivittatus XP_007423311     A..M..K.LK...Y.YSHT.....HISC.K..L..YDQN.C..A.NY.FI
Anolis carolinensis XP_008111248   A..M..K.LK...Y.YSYT.....NITK.KI.L..YDQY.H..V..Y.FI
Chelonia mydas XP_007064264        A.....K.LK...H.F.YT.NIA.H.IR.KIIV...K.S.Y.....F...
Chrysemys picta bellii XP_008171491 A.....K.LK...H.F.YT.NIA.H.IR.KIIF...K.S.Y.....F...
Gallus gallus NP_990382            A.....K.LK...H.F.HM..IA...IR.QIIT...K.S.Y..S..Y...
Meleagris gallopavo XP_010708197   A.....K.LK...H.F.HM..IA...IR.QIII...K.S.Y..S..Y...
Charadrius vociferus XP_009879443  V.R...K.LK...H.F.HI..VA.H.IH.KIIV...K.S.Y....AY...
Cariama cristata XP_009693060      ..R...K.FK...H.F.HM..IA.H.IR.KIIV...K.S.Y.....F...
Egretta garzetta XP_009647475      S.R...K.FK...H.F.HM..IA.H.IR.KIIV...K.S.Y..A..Y...

                                  201                                              250
Consensus                          PSGNXYNIRNLLNEESVLLFTQIKXXRXXXXXXLLETSSPXXXLZXXXHL
Latimeria chalumnae XP_005998517   .A..A.SL...ESK.AI.I...L.GK.QEETE
Xenopus laevis NP_001159485        .P..M..VK.....DA..I.....GGS
Mus musculus NP_031709                    ....H...K....V..S.......R
Elephantulus edwardii XP_006902789 ....Y..........I.......N
Chinchilla lanigera XP_005392894   ....Y...K.......I.......R
Fukomys damarensis XP_010609490    ....Y...K.......I.......R
Canis lupus familiaris XP_005628312 ....Y...K...............R
Felis catus XP_006931147           ....Y...K.......I.......R
Ursus maritimus XP_008706924       ....Y...K...............R
Equus caballus XP_001497371        ....Y...K....K..........R
Pteropus vampyrus XP_011358951     ....Y...K...............R
Orcinus orca XP_012388019          ....Y...................S
Camelus dromedarius XP_010992993   ....Y...................S
Tarsius syrichta XP_008069341      ....Y...K...............R
Homo sapiens AAH41117                     ....Y...K..R............R
Alligator sinensis XP_006027584    .A..A.........D.I.I...L.GE.PVIQDSF
Python bivittatus XP_007423311     .P..I......W.K.C.I....L.GKGQKSNL
Anolis carolinensis XP_008111248   .P..V........K.CII....L.GK.SECE
Chelonia mydas XP_007064264        .E..G...........I.....L.GE.PIIEHS.N.S..S
Chrysemys picta bellii XP_008171491 .E..G...........I.....L.GE.WGK
Gallus gallus NP_990382            .A..G.............H...L.ND.APVGAE.CSVTNENWS.EGKS
Meleagris gallopavo XP_010708197   .A..G.............H...L.NG.RIAGSM.S.P...
Charadrius vociferus XP_009879443  .A..G.................L.KD.TKSKKHM......SLC.QIED..
Cariama cristata XP_009693060      .A..G.................L.KD.PNAGNM.......
Egretta garzetta XP_009647475      .A..G.................L.KD.PKASTS......S

                                  251                           280
Consensus                          VQRNSEEHCNYHHTGNTLQCVVSLILKDLV
Latimeria chalumnae XP_005998517
Xenopus laevis NP_001159485
Mus musculus NP_031709
Elephantulus edwardii XP_006902789
Chinchilla lanigera XP_005392894
Fukomys damarensis XP_010609490
Canis lupus familiaris XP_005628312
Felis catus XP_006931147
Ursus maritimus XP_008706924
Equus caballus XP_001497371
Pteropus vampyrus XP_011358951
Orcinus orca XP_012388019
Camelus dromedarius XP_010992993
Tarsius syrichta XP_008069341
Homo sapiens AAH41117
Alligator sinensis XP_006027584
Python bivittatus XP_007423311
Anolis carolinensis XP_008111248
Chelonia mydas XP_007064264
Chrysemys picta bellii XP_008171491
Gallus gallus NP_990382
Meleagris gallopavo XP_010708197
Charadrius vociferus XP_009879443  ..............................
Cariama cristata XP_009693060
Egretta garzetta XP_009647475
```
