# Supplementary material for: Possible identification of CENP-C in fish and the presence of the CENP-C motif in M18BP1 of vertebrates
Source: F1000Res. 2016 Jan 20;4:474. Originally published 2015 Aug 5. [Version 2] doi: 10.12688/f1000research.6823.2 (PMC4830207; doi:10.12688/f1000research.6823.2)
Supplement: Peer review history

# Peer review: Referee response for version 2

**DOI:** 10.5256/f1000research.8419.r13524

**Published:** 2016-04-22

**Authors:** Inna Lermontova

**Affiliations:** 1 Leibniz Institute of Plant Genetics and Crop Plant Research, Gatersleben, Germany

The CENP-C protein is required for the assembly and proper function of kinetochores in most eukaryotes. In this paper the author has identified the putative CENP-C protein homologue of Teleostei using BLASTP searches. This protein does not contain a typical CENP-C motif, but a motif with a partial homology to CENP-C. However, presence of the Cupin domain at the C-terminus and two CENP-C-like motifs at the central and proximal regions, respectively, have allowed the author to conclude that the identified protein is a CENP-C ortholog.

Additionally, the author has identified the CENP-C motif in M18BP1 proteins of fishes and some other vertebrates. It is interesting that the CENP-C motif cannot be identified in M18BP1 of mammals. From my point of view, this is very important finding of the current study. Therefore, I recommend to show in the paper the structure of the M18BP1 protein homologues of vertebrates with the SANTA, SANT and CENP-C domains. These domains are present in M18BP1 proteins of most vertebrates, but M18BP1 proteins of mammals have only SANTA and SANT domains and in *Latimeria chalumnae* only SANTA and CENP-C domains are present.  I suggest to mention this in the paper and to discuss it.

I think the study is a significant contribution in the field of centromere research and the information presented in the paper is applicable by experimental biologists.

Minor comments:

- I suggest to change the title to: “Identification of the putative CENP-C protein homologue in fish and the CENP-C motif in M18BP1 of vertebrates.”
- Abstract: "[…]but evidence is presented that makes it likely[…]" Please write which evidence.

I have read this submission. I believe that I have an appropriate level of expertise to confirm that it is of an acceptable scientific standard.

# Peer review: Referee response for version 2

**DOI:** 10.5256/f1000research.8419.r12934

**Published:** 2016-04-04

**Authors:** Nikolina Sekulic

**Affiliations:** 1 Department of Chemistry, University of Oslo, Oslo, Norway; 2 Biotechnology Centre, University of Oslo, Oslo, Norway

The paper describes identification of putative CENP-C proteins in fish and points to the presence of CENP-C motif in M18BP1protein of many vertebrates but not mammals.

CENP-C is an essential protein in building centromeres that interacts directly with CENP-A, epigenetic mark of centromeres, and its underlining DNA. Lack of conservation between CENP-C of different species has been attributed to adaptive evolution of the protein. Great evolutional variability has made it difficult to identify CENP-C proteins based only on simple sequence homology search. The author has noticed the lack of annotated CENP-C protein in fish and undertook an effort to use targeted database searches to identify putative CENP-C proteins.

The paper fills in the gap of knowledge in the field.  It presents interesting and valuable findings but I suggest a couple of changes that would help improve it.  

 

1. The central region of the human CENP-C should be labeled as 426-537 and not 426-552. For references see Caroll CW *et al.,* 2010 ^1^; Kato H *et al.*, 2013 ^2^; Falk SJ *et al*., 2015 ^3^, Falk SJ *et al*., 2016 ^4^. 
2. The author couldn’t identify CENP-C motif in the putative proteins in fish but has instead identified a region that is more similar to the central region. Here previous findings on the role of central region and CENP-C motif should be clarified, emphasising that central region specifically binds CENP-A nucleosomes (which targets it to the centromere) (References: Caroll CW *et al.*, 2010 ^1^; Falk SJ e *t al*. 2015 ^3^) while CENP-C motif binds both types of nucleosomes non-selectively (H3 and CENP-A) with lower affinity (Kato H *et al.*, 2013 ^2^). This can be followed with an exception in the chicken system, as suggested by the previous reviewer, Tatsuo Fukagawa.

In this context, I find the absence of CENP-C motif less surprising, since the major function of CENP-C protein (its association with CENP-A nucleosomes) comes from central region anyway – making identified candidate proteins very likely fish CENP-C homologs. Subsequently, identification of the CENP-C motif in M18BP1 proteins is also less surprising knowing that it might just be a nucleosome binding module.   

- In my opinion, region that has similarity to central region should be named “central region-like motif” instead of “cupin-proximal region”.  I find this name very confusing.

 

Minor comments:

 

1. For the more lay audience, specify that teleost is the most populated branch of fish phylogenetic tree
2. In figures 1 and 4, make a distinction between fish and other species, and indicate amino-acid specifications within each species protein like in figures 2 and 3.

I have read this submission. I believe that I have an appropriate level of expertise to confirm that it is of an acceptable scientific standard.

## References

[1] CarrollCWMilksKJStraightAF: Dual recognition of CENP-A nucleosomes is required for centromere assembly. *J Cell Biol*.2010;189(7) : 10.1083/jcb.201001013 1143-55 10.1083/jcb.201001013 20566683PMC2894454

[2] KatoHJiangJZhouBRRozendaalMFengHGhirlandoRXiaoTSStraightAFBaiY: A conserved mechanism for centromeric nucleosome recognition by centromere protein CENP-C. *Science*.2013;340(6136) : 10.1126/science.1235532 1110-3 10.1126/science.1235532 23723239PMC3763809

[3] FalkSJGuoLYSekulicNSmoakEMManiTLogsdonGAGuptaKJansenLEVan DuyneGDVinogradovSALampsonMABlackBE: Chromosomes. CENP-C reshapes and stabilizes CENP-A nucleosomes at the centromere. *Science*.2015;348(6235) : 10.1126/science.1259308 699-703 10.1126/science.1259308 25954010PMC4610723

[4] FalkSJLeeJSekulicNSennettMALeeTHBlackBE: CENP-C directs a structural transition of CENP-A nucleosomes mainly through sliding of DNA gyres. *Nat Struct Mol Biol*.2016;23(3) : 10.1038/nsmb.3175 204-8 10.1038/nsmb.3175 26878239PMC4893769

# Peer review: Referee response for version 2

**DOI:** 10.5256/f1000research.8419.r11994

**Published:** 2016-01-28

**Authors:** Tatsuo Fukagawa

**Affiliations:** 1 Graduate School of Frontier Biosciences, Osaka University, Osaka, Japan

I found that author almost addressed my concerns. The quality of paper has been improved and the paper is of indexable quality.

I have read this submission. I believe that I have an appropriate level of expertise to confirm that it is of an acceptable scientific standard.

# Peer review: Referee response for version 1

**DOI:** 10.5256/f1000research.7336.r10498

**Published:** 2015-09-25

**Authors:** Tatsuo Fukagawa

**Affiliations:** 1 Graduate School of Frontier Biosciences, Osaka University, Osaka, Japan

The centromere protein CENP-C is well known as an essential component for functional kinetochore assembly. Due to importance of this molecule, CENP-C must be conserved in Fish species. The author performed BLASTP searches with the conserved CENP-C motif sequence, but any CENP-C homologues in Fish lineages were not identified with this sequence. However, as there is a putative CENP-C sequence in shark genome, BLASTP searches were carried out with C-terminal domain sequence of putative shark CENP-C. Then, the author identified CENP-C candidates from various teleost genomes. Although the author does not show that candidate proteins localize to centromeres, these candidates contains related sequences of CENP-C motif, which were a little divergent from the vertebrate consensus sequences. Interestingly, the authors found that various vertebrate M18BP1s, which are recruited to centromeres by CENP-C, contain the CENP-C motif sequence, but mammalian homologues do not contain the motif sequence.

This is an observation article and finding of the CENP-C motif in the M18BP1 sequence is interesting. However, to improve the quality of the paper, the author should revise the manuscript. My specific concerns are following.

 

1. As the author recognizes, it is necessary to verify whether CENP-C candidates from teleost genome really localize to Fish centromeres. As the author obtained a candidate from Zebrafish genome, such an experiment is not difficult with the Zebrafish experimental system. If the author added localization data, Figure 2 would be interesting.
2. Related to Figure 2. If the author shows sequence comparison of Cupin domain in teleost sequences, it would be helpful.
3. When the author discuss about central region of human CENP-C, it may be better to cite a recent paper by Nagpa *l et al. *(Mol. Biol. Cell, 2015), which says that central region sequence does not exist in chicken CENP-C. Then, the author can emphasize that the central motif is really important for CENP-A binding.
4. The author described some results of the analysis in the Introduction. This is not necessary and it would be better to cut of redundant description.

I have read this submission. I believe that I have an appropriate level of expertise to confirm that it is of an acceptable scientific standard, however I have significant reservations, as outlined above.

# Response

**Published:** 2016-01-12

**Authors:** Leos Kral

**Affiliations:** University of West Georgia

I have revised the manuscript to address concerns #2, #3 and #4. Unfortunately, I do not have the resources to carry out the localization experiment (concern #1). My main motivation in publishing these observations is to bring awareness of this issue to individuals who may have the resources and interest to follow up with the relevant experiments.
